# Foreign Bodies in the Organs and Tissues of the Body

**Published:** 1846-06

**Authors:** W. B. Herrick

**Affiliations:** Professor of Anatomy in the Rush Medical College


### ARTICLE III.

*Foreign Bodies in the Organs and Tissues of the Body.* By W. B. Herrick, M. D., Professor of Anatomy in the Rush Medical College.

Every experienced practitioner has, doubtless, met with cases with symptoms which, for the want of a correct history, or from inattention to minute circumstances connected with their origin and progress, have appeared inexplicable and perplexing.

Symptoms thus presenting themselves, without any assignable cause, are often produced by the presence of foreign substances imbedded in some important organ or tissue of the body, introduced by accident or otherwise, without the patient's knowledge; its presence not being suspected by himself or his medical attendant.

For the purpose of directing attention to this fact, and to show the importance of inquiring more minutely into the origin and primary cause of such symptoms, we give below the history of a few cases of the kind:

Case I.—In the fall of 1843, I was called to see a Mr. H., an industrious, middle-aged farmer, with a good constitution, who had been suffering, during the 24 hours previous to my arrival, with the most excruciating pain in and around the knee joint, extending upwards to the hip, and downwards to the foot. Limb high-colored, swollen, and very tender, pulse 100 and full.

It appeared, from the history of the case, that while laboring in the field about two months previous to this time, my patient had felt a slight pricking sensation in the integuments covering the joint. Upon examination, a slightly reddened point was discovered, but there being no other evidence of injury, and as exercise caused no inconvenience, he continued his labors up to the time of the inflammatory attack.

Under the influence of antiphlogistic treatment, both general and local, the inflammatory action gradually subsided, and in about ten days all signs of disease had disappeared from the affected part.

About six months subsequent to this attack, being called again, I found Mr. H. suffering as before, with symptoms similar in every respect to those above mentioned. The treatment, this time, though actively antiphlogistic, did not prevent the formation of an abscess in the cellular substance around the joint, which continued to discharge for two weeks, when it healed, leaving no bad effects, apparently, excepting a slightly contracted condition of the muscles of the limb.

In about a year after this second attack, this unfortunate patient was brought upon his bed for the third time, with symptoms identical with the former. An abscess formed as before, which continued to discharge for two or three months, at the end of which time, (during my absence) his medical attendant, while passing a probe into the abscess, discovered a foreign substance imbedded in its walls, which, being withdrawn, proved to be the sharp point of a thorn, a half inch or more in length. After its removal, as may be supposed, the abscess healed kindly, and all traces of disease of the leg and knee rapidly disappeared.

Case II.—A. H., a carpenter, about 25 years of age, of good constitution, and in robust health at the time, was sud denly attacked with cough, profuse expectoration, and difficult respiration, with slight febrile excitement. In the hands of numerous physicians of good reputation, and under the care of as many quacks, for two years after this attack, a part of which time was spent in a hospital at New Orleans, these symptoms became more and more alarming, his sufferings almost insupportable; till at the end of that time, these apparently characteristic symptoms, his emaciated condition and depressed physical powers, impressed the conviction upon himself and medical advisers, that he was about to fall a victim to consumption.

Thus deprived of hope, and desirous of seeing his friends once more, Mr. H. by dint of great exertion, and bodily suffering, arrived at length at the home of his brother in the interior of Illinois, there as he supposed, shortly to end his days.

Soon after his arrival, and during one of the violent fits of coughing, to which he was subject, a foreign substance, which proved to be a fish bone, cuboidal in shape, and a half-inch or more in diameter, was suddenly and forcibly ejected from the laryngial opening upon the floor.

From this time forward, all the alarming symptoms began rapidly to abate, and at this time, two years since, the individual above named, is in perfect health.

After the above fortunate termination of his disease Mr. H. recollected that a month or two previous to the appearance of the above named symptoms, while dining upon fish, he inhaled as he supposed a small portion into the air passages, but as it gave him but little trouble at the time, he thought no more of it, and did not, during his illness, suspect even, the true cause of his sufferings.

Case III.—A friend of mine, a physician, has given me the history of the case of an individual who fell, accidentally, upon the extremity of a blunt stick; which piercing the clothing and integuments, passed into the cellular substance surrounding the lower part of the rectum. The opening thus produced assumed the character of a fistula, and remained open for a long time after the accident. The operation of laying open the cavity, was at length performed, which resulted in the discovery of a piece of cloth imbedded in the tissue at the bottom of the ulcerating canal.

